# Supplementary material for: Data Glove with Self-Compensation Mechanism Based on High-Sensitive Elastic Fiber-Optic Sensor
Source: Polymers (Basel). 2022 Dec 26;15(1):100. doi: 10.3390/polym15010100 (PMC9824815; doi:10.3390/polym15010100)
Supplement: Supplementary file 1 [file polymers-15-00100-s001.zip › Supplementary File.pdf]

1.All the components of this data glove were purchased at a very cheap price. The actual price of each component is recorded in Table S1.

Components	Price
Textile gloves	$4.4
Cameras and light sources	$11.6
Materials for fabricating flexible optical fibers	$5.8
Total sum	$21.8 (Material cost, Labor costs are not included)

Table S1. The actual price of each component of the data glove.

2. **Measurements.**

In order to make the measurement method easy to observe, we have made physical pictures to describe the measurements. As shown in Figure S1, iron bars of different radii are used to adjust the bending radius of the elastic fiber. Radius of iron bar increased from 2.5mm to 10mm. The optical loss of the fiber is then measured at different bending radii.

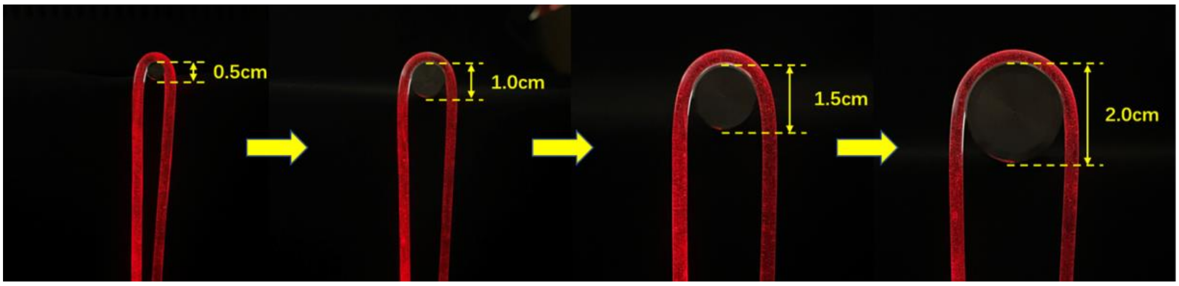

Figure S1. Elastic fiber with different bending radius.

Figure S2 shows the measurement process of the maximum stretching range of U-shaped fiber. Increase the stretch at 10% intervals in the sequence from Figures S2a to S2e until extinction occurs. The normalized spectrum of the fiber in each stretch state is then measured.

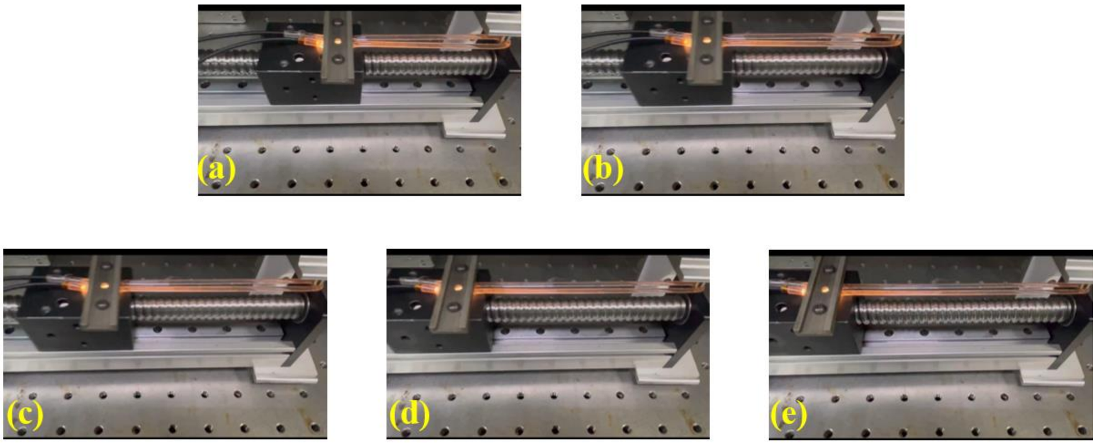

Figure S2. The measurement process of the maximum stretching range of U-shaped fiber.

The indentation test process is shown in Figure S3. We place the tension meter perpendicular to the side of the fiber and increase the force successively from 0N to 100N at 10N intervals. The optical loss in each state of the fiber is then measured.

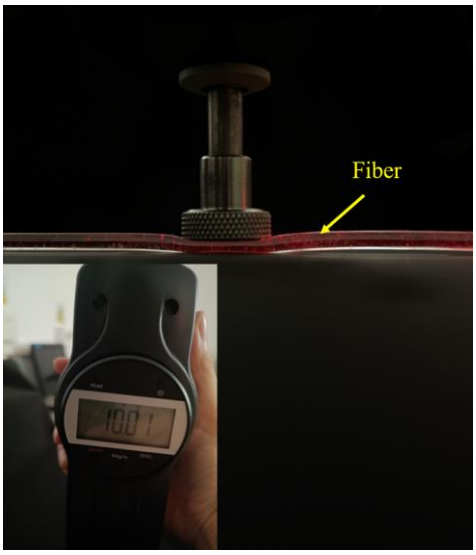

Figure S3. The physical picture of the indentation test.

Figure S4 shows the bending cycle test. We define an optical fiber as a cycle from a straight line state to a half fold state and then back to a straight line state(from Figure S4a to S4g and then back to Figure S4a). Repeat the cycle 100 times and measure the normalized spectrum of the fiber after each cycle.

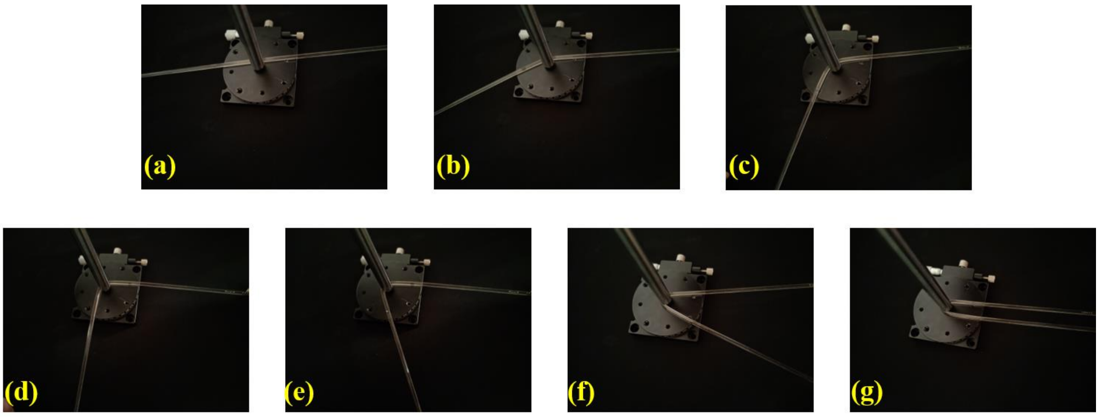

Figure S4. The definition of a cycle in a bending cycle test.

For the stretch cycle test, please watch video S1 in the supplementary materials. Tensile cycle test at different temperatures is carried out by placing the equipment of tensile cycle test in an incubator.
